# Late Pleistocene/Early Holocene Migratory Behavior of Ungulates Using Isotopic Analysis of Tooth Enamel and Its Effects on Forager Mobility

**DOI:** 10.1371/journal.pone.0155714

**Published:** 2016-06-08

**Authors:** Suzanne E. Pilaar Birch, Preston T. Miracle, Rhiannon E. Stevens, Tamsin C. O’Connell

**Affiliations:** 1 Department of Anthropology and Department of Geography, University of Georgia, Athens, Georgia, United States of America; 2 Department of Archaeology and Anthropology, University of Cambridge, Cambridge, United Kingdom; 3 Institute of Archaeology, University College London, London, United Kingdom; 4 McDonald Institute for Archaeological Research, University of Cambridge, Cambridge, United Kingdom; New York Institute of Technology, UNITED STATES

## Abstract

Zooarchaeological and paleoecological investigations have traditionally been unable to reconstruct the ethology of herd animals, which likely had a significant influence on the mobility and subsistence strategies of prehistoric humans. In this paper, we reconstruct the migratory behavior of red deer (*Cervus elaphus*) and caprids at the Pleistocene-Holocene transition in the northeastern Adriatic region using stable oxygen isotope analysis of tooth enamel. The data show a significant change in δ^18^O values from the Pleistocene into the Holocene, as well as isotopic variation between taxa, the case study sites, and through time. We then discuss the implications of seasonal faunal availability as determining factors in human mobility patterns.

## Introduction

The seasonal availability of plant and animal resources was integral to the subsistence and mobility strategies of past human groups [1–6] and the migratory behavior of large herbivore species has long been used as a proxy for the mobility of late Pleistocene hunter-gatherers in Europe, relying on modern ethology as a baseline for interpretation (e.g. Paleolithic Epirus, Greece, [7–10]). Since the end of the last ice age, substantial changes in landscape, climate, and human activity have influenced habitat size, vegetation, and population levels. It is therefore likely that significant changes in herbivore mobility, including preferred pathways as well as range distance, have occurred. Seasonal predictability and reliability of these faunal resources may have been crucial for human survival at times of environmental change or climatic instability.

This study investigates the mobility of herbivore prey species at the head of the Adriatic (Istria, Croatia) during the Pleistocene-Holocene transition (12,000–8,000 years ago) and spans the cultural phases of the Late Upper Paleolithic, the Mesolithic, and the early Neolithic. Zooarchaeological evidence attests to the economic importance of ungulate taxa including red deer (*Cervus elaphus*), ibex (*Capra ibex*), and chamois (*Rupicapra rupicapra*) at a number of cave sites in the region throughout time [11–17]. We use intra-tooth stable isotope data from archaeological specimens at three of these cave sites to test assumptions about the prehistoric migratory behavior of these prey species based on modern ethology.

The present day migratory behavior of red deer is variable, with movement between habitats due to differences in seasonal forage quality, weather, pests, mating, and current wildlife management practices [18–23]. In addition, adjacent populations of red deer may have very different migration and mobility patterns [18, 24] over variable distances (e.g., from 10–140 km [23]). Migratory behavior is most often described in cold-adapted populations of North American elk (*Cervus elaphus canadensis*) e.g. [18, 19, 23], which offers a potential analogue for red deer populations extant during the cold climate of the terminal Pleistocene in Europe. If the mobility of this species is predominantly determined by environmental factors in the present, there must be a substantial amount of uncertainty in predicting expected mobility patterns in the past, and red deer may or may not have been migratory depending on specific local conditions. This would have had implications for the lifestyles of hunter-gatherers in the northeastern Adriatic during the late Pleistocene, whose specialized subsistence economy was based on red deer [12–17].

Though most caprids are traditionally regarded as high-altitude taxa, this may be a result of present-day distribution of steep, craggy rock faces at high altitudes rather than an altitudinal preference [25] and archaeological evidence from the study region suggests that they occupied elevations much lower than this (100m above sea level) in the late Pleistocene [26, 27]. Caprids do not migrate laterally across large distances, and most of their movement is related to mating and forage quality (*R*. *rupicapra*, [28–29]; *R*. *pyrenaica* [30–31]; *C*. *pyrenaica* [32]). This suggests that populations of chamois and ibex in the past were likely not seasonally migratory over long distances, making them a more accessible resource throughout the year. Based on the faunal data at the case study sites [15–17] there is a shift away from the late Pleistocene hunting of red deer-whose modern day behavioral ecology suggests are selective migrators-to multiple species, including chamois and ibex, which are assumed to be non-migratory, in the early Holocene.

Testing whether the migratory behavior seen in modern populations occurred in the past remains a challenge using traditional zooarchaeological methods. Stable isotope analysis has many applications to zooarchaeological inquiry, contributing to topics such as diet, climate, ecology and mobility in the past [33]. Here we seek to establish the nature of seasonal mobility of red deer and caprids using the incremental analysis of δ^18^O from their tooth enamel carbonate. The intra-tooth δ^18^O isotopic variation of an animal with limited mobility reflects seasonal variations in meteoric water δ^18^O, which in high and mid latitudes tracks ambient temperature. Animals that move between summer and winter pastures throughout the year along latitudinal or altitudinal gradients minimize the seasonal variation in their external environment, resulting in a damped isotope signal and lower intra-tooth variability. Thus a larger range in δ^18^O values are expected to be seen within a single tooth of an animal with limited mobility than in a migratory animal.

A cyclical oxygen isotopic pattern has been well-documented in ovicaprids, domestic sheep (*Ovis aries*) and goat (*Capra hircus*) [34–36] and observed in a non-migratory red deer population in Scotland [37]. The observed range in δ^18^O for non-migrating individuals is 2–4‰ for caprids [34] and between 3–4‰ for red deer [37]. In contrast, damped oxygen and carbon isotopic fluctuations in tooth enamel have been shown in migratory modern North American caribou (*Rangifer tarandus*) with a low range of intra-tooth variation (approximately 1‰ in δ^18^O) [38]. If migratory, the archaeological red deer in this study should exhibit limited intra-tooth isotopic variability; if not, seasonal intra-tooth isotopic variation should be detectable. Likewise, non-migratory caprids in this study are expected to exhibit relatively higher intra-tooth isotopic variation.

### Environment and archaeology of the study region

**Fig 1:**
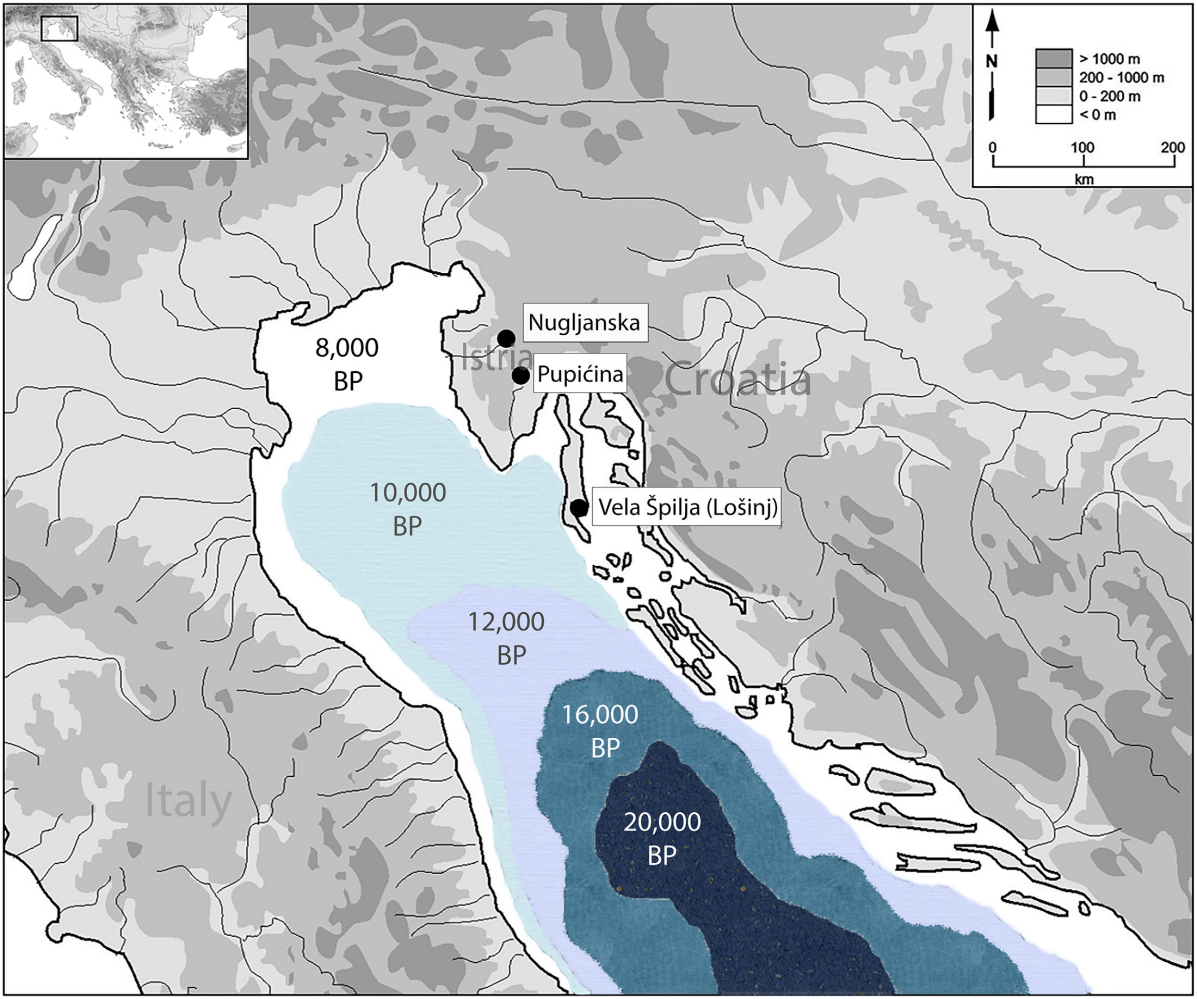
Map of the study region showing sampled sites.

**Fig 2:**
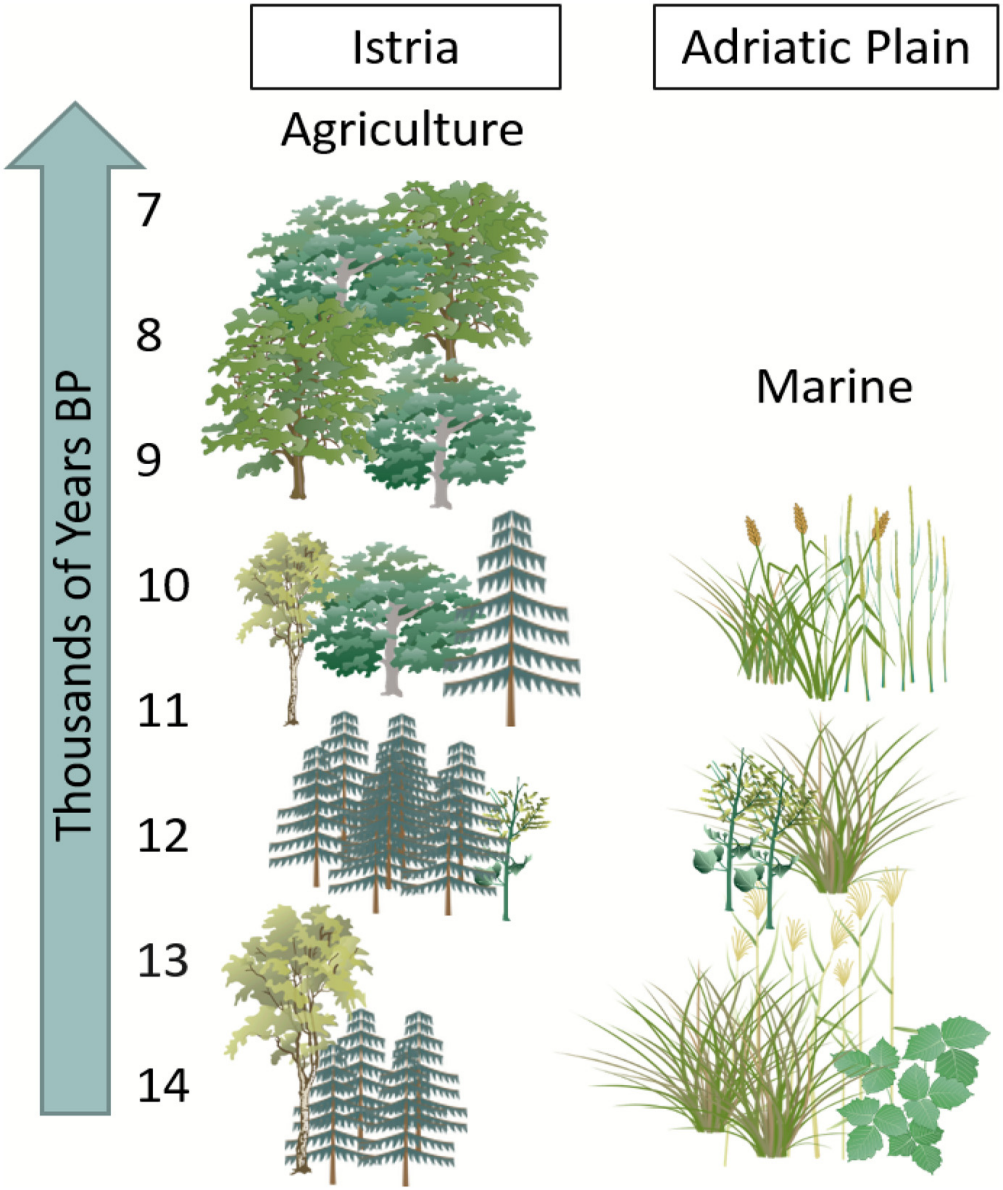
Schematic of vegetation change on Istria and the Great Adriatic Plain through time. Clip art from [98].

The Istrian Peninsula of Croatia has a diverse topography and coastline, unique environmental history, and is a “crossroads” location between Europe and Southwest Asia. Based on regional records, the terminal Pleistocene Bølling-Allerød interstadial (14,600–12,900 BP) was characterized by a sustained rise in temperatures and increased water availability, leading to the spread of forest into upland areas from refugial populations located on lower slopes in Istria and the surrounding area [14, 39–42]. The Younger Dryas (12,900–11,500) was cool and dry, and grassland and open shrubland were the dominant groundcover [42–45]. In the initial Holocene, warming resumed in the Pre-Boreal and Boreal (11,500–9,000 BP) and the area became reforested with deciduous species [46–48]. There was some variability in vegetation because of the short-lived cooling oscillations within these periods, perhaps creating a “patchy” environment [45]. The Atlantic period (9,000–6,000 BP) was warmer and wetter, and the arrival of agriculture in the region at this time significantly altered the vegetative cover as land was cleared and new species introduced [41]. These climate-driven environmental and ecological changes would have strongly influenced human settlement patterns, seasonal site use, and subsistence strategies at the Pleistocene-Holocene transition. In addition, rapid sea level rise would have played a critical role in shaping the landscape. Following deglaciation, the level of the Adriatic Sea rose rapidly, but not at a constant rate; sea level was -23m by 8,500 years ago when the area first became submerged by freshwater (Fig 1) [49, 50]. Changes in regional ecosystems as the Great Adriatic Plain disappeared and habitat transition from grassland to coastal environments (Fig 2) were likely a key factor in influencing the seasonal mobility of large herbivores that were a staple resource at this time.

There is evidence of late Pleistocene/Late Upper Paleolithic and early Holocene/Mesolithic forager groups using cave sites in the Istrian Peninsula as hunting outposts and base camps where they made tools and may have held ritual feasts [12, 51]. In contrast, Late Upper Paleolithic and Mesolithic open air sites at lower elevations are rare. This dearth of archaeology could represent the reality of an uninhabited landscape, but is not due to lack of survey and research [51]. The most likely explanation is the loss of sites due to the inundation of the Great Adriatic Plain, which may well have been the center of human population during the Paleolithic until the incursion of the sea in the terminal Pleistocene. In this case, Istrian cave sites can be placed in the hinterland, which was only explored towards the end of the Late Upper Paleolithic and during the Mesolithic period [52–53]. Neolithic occupation appears to be primarily by agro-pastoralists using the caves for herding activities [54–56].

### Stable isotope analysis and modelling migration

The δ^18^O signature recorded in tooth enamel carbonate in mammals is related to meteoric δ^18^O_water_ [57–60], which can be related to temperature [61, 62], such that tooth enamel δ^18^O_carbonate_ can be used as a proxy for temperature. In temperate environments, the δ^18^O_water_ values are higher in warm temperatures and lower in cooler temperatures [63]. Due to the nature of tooth mineralization processes in hypsodont teeth, in temperate climates the intra-tooth δ^18^O signal varies with seasonal fluctuations in temperature and records sub-annual temperature variability c.f. [64–67] although the tooth enamel isotopic signal is modulated by a number of factors, including the temperature—water isotope relationship, the available water sources, species-specific physiological effects [68, 69] tooth mineralization rates [64, 66, 70] and sampling [65,71]. Damping of the δ^18^O signal occurs to a varying degree depending on the animal’s subfamily and genera, c.f. [72]. In caprids, this damping factor is approximately 10% [35, 64, 73, 74] and can be up to 50% in cervids [72].

Tooth enamel carbonate δ^13^C values are reflective of carbon isotopic values of the whole diet with herbivore bioapatite δ^13^C values higher than the diet by 12–14‰ [64, 75, 76], particularly in ruminants [77, 78]. In addition to isotopic fractionation that occurs during photosynthesis [79], external factors such as water availability, temperature [80], light [81], salinity [82–84], and altitude [85–88] influence carbon isotopic ratios in the plant and will affect the eventual δ^13^C signal in herbivores. Observed differences in δ^13^C values in intra-tooth samples can be ascribed to changes in diet during tooth formation such as browsing rather than grazing and, as noted above, may be affected by the rate of tooth mineralization in different species [64, 66, 70]. For these reasons, faunal carbon isotopic values are not used for reconstructing seasonal migration in this paper, but are included as an indicator of diet rather than a measure of variability.

## Materials & Methods

### Selection of Samples

Ten red deer and fourteen caprid teeth were sampled from the sites of Pupićina (PUP), Nugljanska (NUG), and Vela Špilja Lošinj (VSL) (c.f. Fig 1). Samples were selected based on tooth (M2 or M3), tooth wear (less wear is desirable) and taxonomic identification. Verification that teeth from the same stratigraphic context were not from the same individual was based on ontogenetic age, symmetry, wear, coloration, and morphology of the teeth. At least two specimens of each taxon were selected from each stratigraphic horizon when possible. Balasse and colleagues [36] recently suggested based on the M2 in sheep that teeth should be at least 18mm in height (half the average height of the tooth) in order to record the maximum and minimum δ^18^O over an annual cycle. The dental morphology of ovicaprids, which includes sheep, goat, chamois, and ibex, are similar such that this expectation can be applied to these taxa. Twelve out of fourteen caprid teeth meet or exceed the suggested height of 18mm. A minimum sampling height for *Cervus elaphus* has not been established. However, to record the maximum and minimum δ^18^O over an annual cycle sampling should go across M2-M3 pairs as these teeth mineralize over a period of nine months each and grow sequentially [37, 89]. As only isolated red deer teeth were available for sampling, M3 were selected wherever possible. Contextual information for isotopically sampled teeth is presented by site in Table 1. The distinction of chamois (*Rupicapra rupicapra*) and ibex (*Capra ibex*) from one another is problematic, similar to that of domestic sheep (*Ovis aries*) and goats (*Capra hircus*). While there has been much literature devoted to distinguishing sheep and goats through osteology and tooth morphology [90–95] there is a lack of sources dedicated to distinguishing chamois and ibex from each other. But although difficult to distinguish osteologically (especially when highly fragmented), separating chamois and ibex may not make much of a difference for paleoecological reconstructions. They fill a very similar ecological niche, have similar behaviors, provide similar amounts of meat, and have similar modern, and perhaps prehistoric, ecomorphology [28, 30, 31].

**Table 1:** Contextual information for teeth selected for isotopic analysis.

Lab ID	Specimen ID	Radiocarbon ID	Status	Taxon	Period	Level	^14^C cal BP	Tooth	Crown Height (mm)
**NUG 1**	12.072	OxA 26060	Destroyed	*Cervus*	Mesolithic	4	8,770–9,020*	LM_3_	25
**NUG 2**	12.191		Returned	*Cervus*	Mesolithic	4	8,980–9,316	RM_3_	19
**NUG 3**	12.249		Returned	Caprid	Mesolithic	4	8,980–9,316	LM_2_	16
**NUG 6**	17.429	OxA 26347	Destroyed	*Cervus*	Mesolithic	5	8,716–9,007*	LM_3_	18
**NUG 11**	14.116	OxA 26059	Destroyed	Caprid	Mesolithic	5	9,345–9,400*	RM_3_	20
**NUG 13**	18.467		Returned	*Cervus*	Paleolithic	6	12,845–13,225	LM_3_	25
**NUG 17**	20.088	OxA 2462–26	Destroyed	*Cervus*	Paleolithic	6	12,845–13,225*	RM_3_	27
**NUG 24**	23.040		Returned	Caprid	Palaeolithic	7	>13,225	RM^2^	27
**NUG 25**	28.020	OxA 2462–22	Destroyed	Caprid	Palaeolithic	8	14,212–15,077*	RM^2^	40
**VSL 5**	65.080		Returned	Caprid	Neolithic	45	6,955–7,170	RM_3_	32
**VSL 6**	65.078	OxA 26174	Destroyed	Caprid	Neolithic	45	6,955–7,170*	RM_3_	29
**VSL 1**	19.001	OxA 32823	Destroyed	Caprid	Meso/Neolithic	60	7,011–7,252*	RM^3^	32
**VSL 2**	69.003	OxA 26173	Destroyed	Caprid	Meso/Neolithic	60	7,150–7,259*	RM^3^	25
**PUP 3**	609.38		Returned	Caprid	Neolithic	331	6,890–7,425	LM_3_	30
**PUP 4**	581.26		Returned	Caprid	Neolithic	324	6,890–7,425	LM_3_	28
**PUP 7**	892C.17		Returned	Caprid	Mesolithic	346	10,385–11,400	LM_2_	20
**PUP 8**	892C.17		Returned	Caprid	Mesolithic	346	10,385–11,400	LM_3_	17
**PUP 28**	758A.34		Returned	Cervid	Mesolithic	345	9,905–10,820	LM_3_	19
**PUP 29**	766B.7		Returned	Cervid	Mesolithic	345	9,905–10,820	LM_3_	21
**PUP 31**	226B.18		Returned	Cervid	Mesolithic	202	9,905–10,820	LM_3_	24
**PUP 24**	1256B.511		Returned	Cervid	Paleolithic	361	11,950–12,960	LM_2_	18
**PUP 26**	1259.567		Returned	Cervid	Paleolithic	354	11,950–12,960	LM_2_	15
**PUP 12**	1277B.807		Returned	Caprid	Paleolithic	363	>12,960	LM_2_	40
**PUP 15**	1287B.807		Returned	Caprid	Paleolithic	363	>12,960	RM_2_	27

An asterisk (*) indicates that the tooth was directly dated. Dates for Nugljanska are previously published [15]. Dates for VSL are published here for the first time. “Older than” estimates are provided for undated layers (Nugljanska Level 7, Pupićina Horizon R2).

Though sample sizes are limited due to the nature of the archaeological record, when teeth are sub-sampled as few as 4 samples can be drilled at equal increments along the cusp to calculate mean δ^18^O with a standard deviation of ±0.01‰ at 95% confidence in a sample as small as 4 teeth [96]. At least 3 teeth, and optimally 5–6, should be sampled for each stratigraphic level when making comparisons between groups [97]. We have sampled at least 4 teeth from each phase, and generally taken 8–10 subsamples per tooth.

### Preparation and Analysis

Once measured and photographed, teeth were drilled along the growth axis in the direction of tooth formation, from the crown to cervix, using a Marathon dental drill and a 1mm diamond-tipped drill bit. Powdered enamel was transferred into pre-weighed tubes, which were then weighed again in order to calculate the sample weight (between 3–4 mg) prior to cleaning. Samples from Nugljanska and Vela Špilja Lošinj were drilled and prepared at the Dorothy Garrod Laboratory, McDonald Institute for Archaeological Research, University of Cambridge. Samples from Pupićina were drilled at the Arheološki Muzej Istre in Pula, Croatia or in Cambridge and were prepared in the Dorothy Garrod Laboratory. Specimens were sampled with permission from the Arheološki Muzej Istre (Pula) and the Croatian Academy of Sciences and Arts (Zagreb). Teeth that were radiocarbon dated at Oxford University Radiocarbon Accelerator Unit were destroyed in the sampling process; all other specimens have been returned to archaeological collections held at the Arheološki Muzej Istre.

After being drilled, enamel powder was treated for bioapatite extraction using the method described in Balasse and colleagues 2002 [34]. First, 2% aq. sodium hypochlorite solution was added to the samples to remove organic matter, followed by 0.1M aq. acetic acid to remove exogenous carbonate, and then freeze dried. Additional matrix-matched tooth enamel standards were included within each batch of samples to ensure sample fidelity and to rule out contamination during the sample preparation process. Samples were isotopically analyzed at the Godwin Laboratory, Department of Earth Sciences, University of Cambridge. Samples from Pupićina and VSL were reacted with 100% orthophosphoric acid at 90°C using a Micromass Multicarb Preparation System, then the carbon dioxide produced was dried and transferred cryogenically into a VG SIRA mass spectrometer or VG PRISM mass spectrometer. Samples from Nugljanska were analyzed on a Thermo Finnigan MAT253 coupled with a Gas Bench II. Based on repeat measurements of isotopic standards, the precision is better than ±0.08‰ for δ^13^C and better than ±0.10‰ for δ^18^O for machine standards. Results are reported with reference to VPDB and expressed as δ^18^O ‰ = [R(sample)/R(standard)]-1·1000. All statistics were computed using the Paleontological Statistics (PAST) program [99]. The data were found to be normally distributed using a Shapiro-Wilk test or were otherwise large enough under central limit theorem to apply parametric tests. This included a Student’s t-test to determine whether mean values and variances of teeth for cervid and caprid sample groups were significantly different between the late Pleistocene/Late Upper Paleolithic and early Holocene/Mesolithic periods.

## Results

### Ranges of variation in δ^18^O and δ^13^C through time

Isotopic data from each individual are summarized in Table 2. In general, caprids in all archaeological levels at the case study sites have a larger intra-tooth δ^18^O and δ^13^C range than do red deer. These differences are here argued to be attributable to species-specific migratory and foraging behaviors. Species and individual physiology, as well as crown height and period of tooth formation, may also contribute to variation. Below, we consider variability within groups of caprids and red deer through time and between species.

**Table 2:** Minimum, maximum, range, and mean values of δ^18^O and δ^13^C from caprid and red deer teeth at the case study sites.

Sample	Taxon	Period	^14^C cal BP	Tooth	Crown Height (mm)	Min δ^18^O	Max δ^18^O	Range δ^18^O	Mean δ^18^O	Min δ^13^C	Max δ^13^C	Range δ^13^C	Mean δ^13^C
**NUG 24**	Caprid	Paleolithic	>13,225	RM^2^	27	-6.9	-3.0	3.9	-5.4	-11.6	-11.0	0.6	-11.3
**NUG 25**	Caprid	Paleolithic	14,212–15,077*	RM^2^	40	-8.4	-2.6	5.8	-5.7	-12.1	-9.7^x^	2.4	-11.5
**PUP 12**	Caprid	Paleolithic	>12,960	LM_2_	40	-9.0	-3.7	5.3	-6.3	-10.7	-5.7^x^	5.0	-9.4
**PUP 15**	Caprid	Paleolithic	>12,960	RM_2_	27	-8.9	-4.4	4.4	-6.9	-11.0	-9.7	1.3	-10.3
**PUP 7**	Caprid	Mesolithic	10,385–11,396	LM_2_	20	-6.3	-1.5	4.8	-4.3	-11.6	-9.5	2.1	-11.0
**PUP 8**	Caprid	Mesolithic	10,385–11,396	LM_3_	17	-6.1	-3.3	2.9	-5.0	-11.3	-8.8	2.4	-10.5
**NUG 11**	Caprid	Mesolithic	9,345–9,402*	RM_3_	20	-5.7	-1.5	4.2	-3.4	-10.8	-10.2	0.7	-10.4
**NUG 3**	Caprid	Mesolithic	8,977–9,316	LM_2_	16	-2.7	-0.2	2.5	-1.3	-12.2	-10.3	1.9	-11.1
**VSL 1**	Caprid	Meso/Neolithic	7,561–7,665*	RM^3^	32	-5.0	-2.4	2.5	-3.7	-11.9	-6.4	5.5	-9.0
**VSL 2**	Caprid	Meso/Neolithic	7,150–7,259*	RM^3^	25	-6.2	-1.1	5.1	-2.9	-12.5	-9.4	3.1	-10.9
**VSL 5**	Caprid	Neolithic	6,955–7,170	RM_3_	32	-4.8	-1.4	3.4	-3.3	-12.0	-10.1	1.8	-11.0
**VSL 6**	Caprid	Neolithic	6,955–7,170*	RM_3_	29	-4.5	-1.3	3.2	-2.8	-12.6	-9.8	2.8	-11.1
**PUP 3**	Caprid	Neolithic	6,890–7,425	LM_3_	30	-5.9	-3.2	2.7	-5.0	-13.6	-9.2	4.4	-11.0
**PUP 4**	Caprid	Neolithic	6,890–7,425	LM_3_	28	-7.9	-4.1	3.8	-6.3	-12.7	-11.2	1.5	-12.1
**NUG 13**	*Cervus*	Paleolithic	12,845–13,225	LM_3_	25	-7.8	-5.9	2.0	-7.0	-12.1	-11.3	0.8	-11.7
**NUG 17**	*Cervus*	Paleolithic	12,845–13,225*	RM_3_	27	-7.2	-6.2	0.9	-6.9	-11.9	-10.5	1.3	-11.4
**PUP 26**	*Cervus*	Paleolithic	11,950–12,960	LM_2_	15	-8.0	-6.3	1.7	-7.1	-11.4	-10.1	1.4	-10.9
**PUP 24**	*Cervus*	Paleolithic	11,950–12,960	LM_2_		-7.7	-7.4	0.4	-7.6	-12.9	-11.3	1.6	-12.2
**PUP 28**	*Cervus*	Mesolithic	9,905–10,820	LM_3_	19	-6.9	-4.6	2.3	-6.0	-12.2	-8.0	4.2	-10.1
**PUP 29**	*Cervus*	Mesolithic	9,905–10,820	LM_3_	21	-7.8	-5.6	2.3	-6.7	-14.2	-10.3	3.9	-12.6
**PUP 31**	*Cervus*	Mesolithic	9,905–10,820	LM_3_	24	-5.8	-3.3	2.4	-4.7	-11.7	-10.0	1.6	-11.1
**NUG 6**	*Cervus*	Mesolithic	8,716–9,007*	LM_3_	18	-7.4	-5.1	2.3	-6.6	-13.2	-12.6	0.7	-12.9
**NUG 2**	*Cervus*	Mesolithic	8,977–9,316	RM_3_	19	-7.7	-5.4	2.4	-6.6	-13.3	-12.7	0.6	-13.1
**NUG 1**	*Cervus*	Mesolithic	8,769–9,024*	LM_3_	25	-7.4	-4.6	2.8	-5.6	-12.4	-11.8	0.6	-12.1

Dates marked with an asterisk (*) denote samples where the tooth was directly dated. The two δ^13^C maximum values marked with an (^x^) (NUG 25 and PUP 12) were identified as outliers within the text. All δ values are in per mil VPDB.

### Variation within Caprids through Time

**Fig 3:**
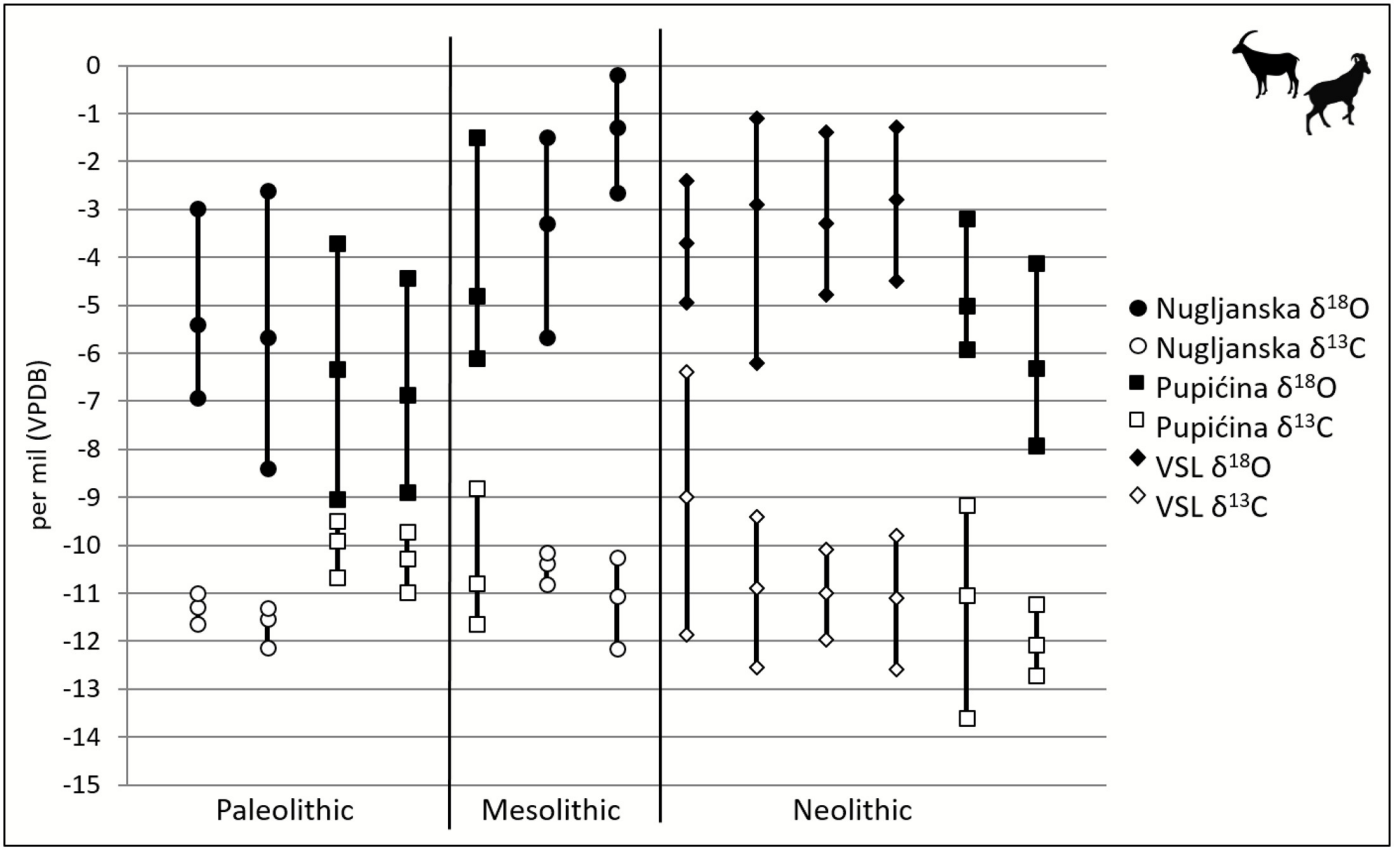
Oxygen and carbon isotopic values of caprid individuals by phase. Each vertical line represents an individual (e.g., NUG 25), with points representing mean, minimum and maximum.

The fourteen caprid teeth from Pupićina, Nugljanska, and VSL span the Late Upper Paleolithic (terminal Pleistocene) and Mesolithic and Neolithic (early Holocene), and represent 13 individuals (the teeth PUP 7 and PUP 8 are from one individual). When the carbon and oxygen isotopic data from each individual are plotted by period, a few temporal patterns emerge (Fig 3). First, there is a significant (Student’s t-test, p<0.001) increase in δ^18^O from the Late Upper Paleolithic (4 individuals, n = 46, overall mean δ^18^O = -6.8‰) to the Mesolithic period (3 individuals, n = 27, overall mean δ^18^O = -3.2‰). This is greater than the ~1‰ difference between Late Pleistocene and Holocene global ocean δ^18^O, suggesting this increase does not solely reflect a change in the δ^18^O of source waters and likely reflects increasing temperatures. This supports paleoclimatic reconstructions that suggest a regional warming trend from the Pleistocene to the Holocene, as summarized above. While the mean δ^18^O is slightly lower in the Neolithic samples (6 individuals, n = 54, overall mean δ^18^O = -3.9‰) relative to those from the Mesolithic, this difference is not significant (Student’s t-test, p = 0.1). The wide intra-tooth variation in caprid oxygen isotope values indicates that these individuals recorded a large range of temperatures in their dental enamel in all time periods. Both the intra-tooth and inter-individual δ^13^C results for caprids are more variable in the Neolithic than in earlier periods, but there is no significant change of mean δ^13^C values through time for caprids.

### Variation within Red Deer through Time

**Fig 4:**
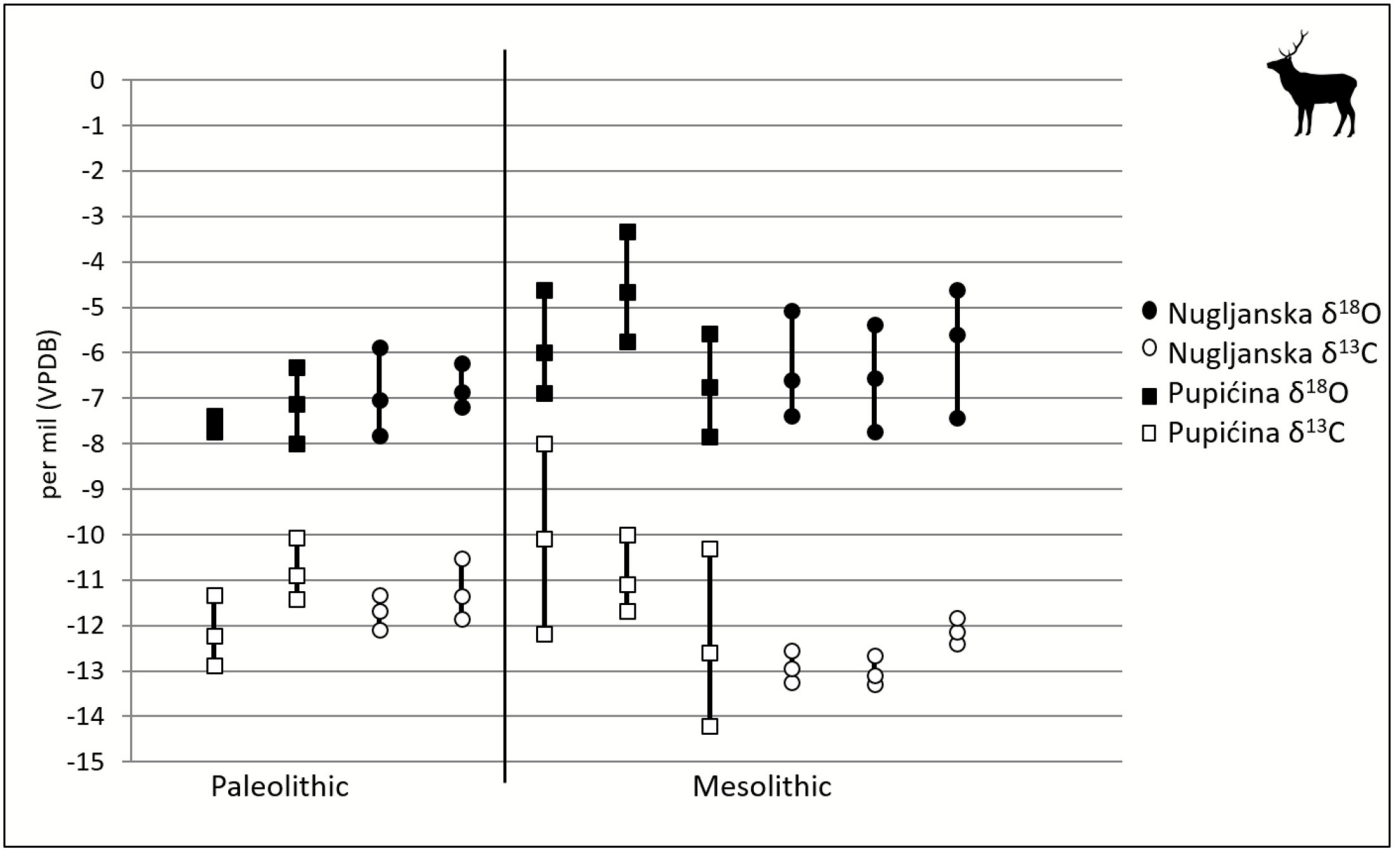
Oxygen and carbon isotopic values of red deer individuals by phase. Each vertical line represents an individual (e.g., NUG 13), with points representing mean, minimum and maximum, n = 86.

Ten red deer teeth from ten individuals were analyzed from Late Upper Paleolithic (terminal Pleistocene) and Mesolithic (early Holocene) levels at Nugljanska and Pupićina (Fig 4). There is a significant difference (Student’s t-test, p<0.001) in δ^18^O values between the 4 individuals analyzed in the Late Upper Paleolithic (mean δ^18^O = -7.1‰, n = 27) and the 6 individuals in the Mesolithic (mean δ^18^O = -6‰, n = 59). The increase in variance in δ^18^O values from the Late Upper Paleolithic to the Mesolithic is significant (Student’s t-test, p = 0.001). There is also a significant difference (Student’s t-test, p = 0.03) in δ^13^C between the 4 individuals sampled from the Late Upper Paleolithic (mean δ^13^C = -11.2‰, n = 27) and the 6 individuals in the Mesolithic sample (mean δ^13^C = -11.9, n = 59). The range of carbon isotope values also appears to be larger in the Mesolithic group, but this increase in variability is not statistically significant.

### Variation in Caprids vs. Red Deer

**Fig 5:**
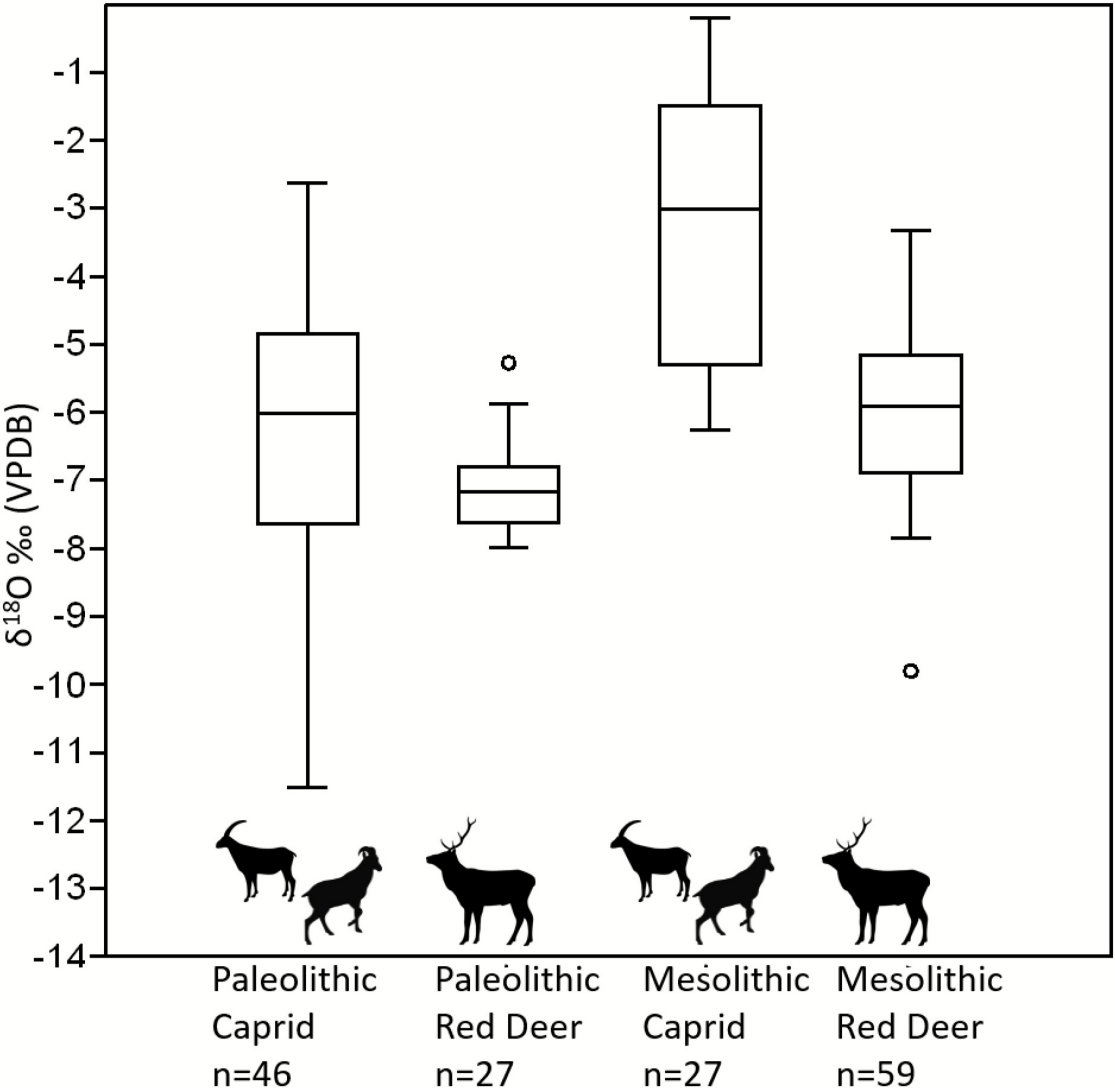
Boxplot of δ^18^O values in caprids and red deer through time, where box = 50% and whiskers are 95%.

There is less intra-species variation in enamel oxygen isotope values for red deer than for caprids in both the Late Upper Paleolithic and Mesolithic (Fig 5). The mean δ^18^O based on all measured values for the Late Upper Paleolithic caprids was -6.2±1.9‰, whereas the mean δ^18^O for red deer during this time was -7.1±0.6‰. Both the mean values and variances of these taxa are significantly different (Student’s t-test, p<0.001). The mean δ^18^O based on all measured values for Mesolithic caprids was -3.2±2‰, whereas the mean δ^18^O for red deer in this period was -6±1.1‰. The means and variances for these two taxa are significantly different (Student’s t-test p<0.001) in both periods.

**Fig 6:**
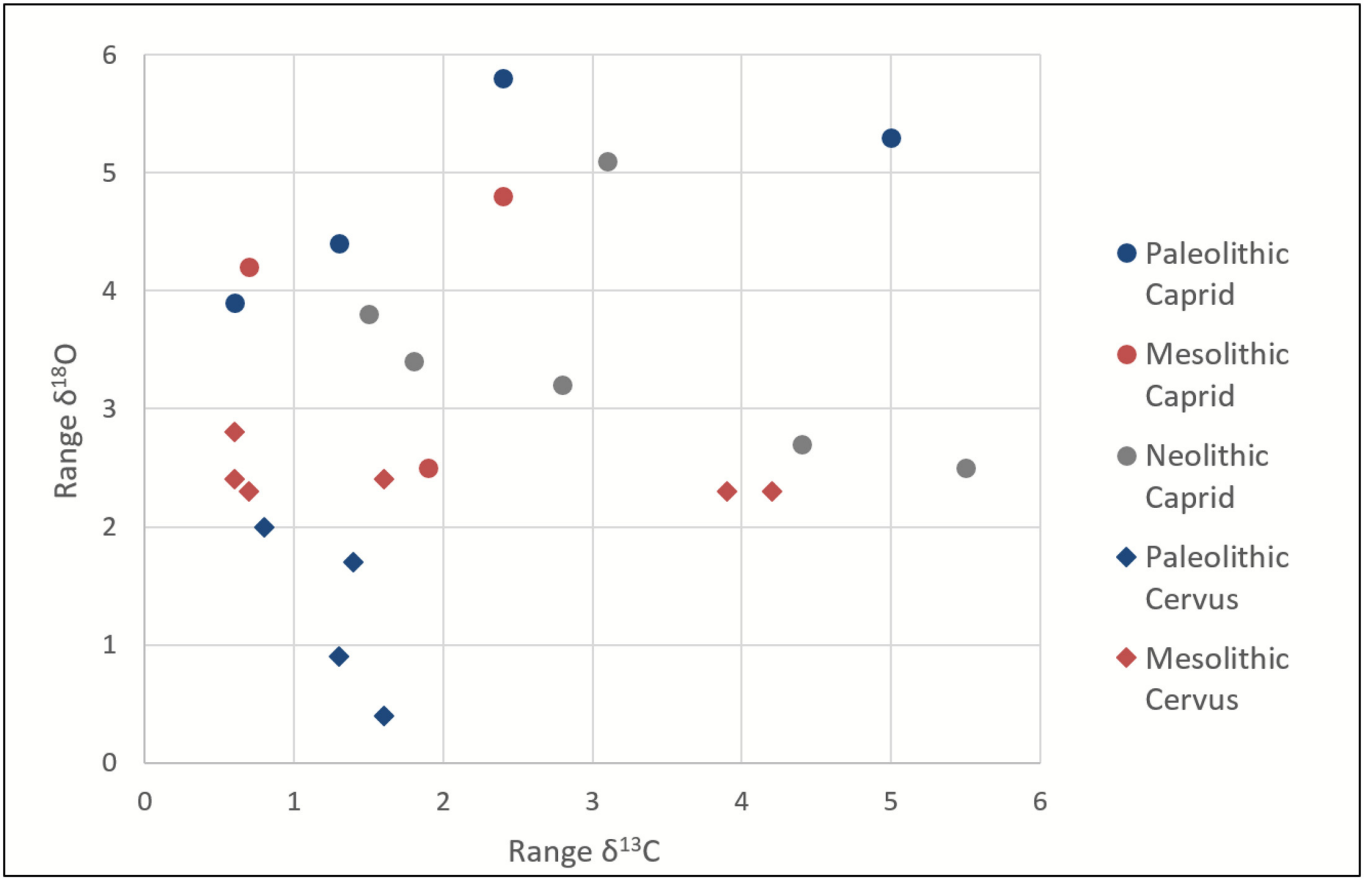
Scatter plot of intra-individual ranges of δ^18^O and δ^13^C values, which shows an increase in intra-individual variability of δ^18^O and no change in δ^13^C in *Cervus* specimens from the late Pleistocene/Paleolithic to the early Holocene/Mesolithic and no pattern in intra-individual variability of δ^18^O or δ^13^C in caprids between the late Pleistocene/Paleolithic and the early Holocene/Mesolithic and Neolithic.

Intra-individual carbon and oxygen isotopic variation differs by species over time (Fig 6). For Late Upper Paleolithic *Cervus* intra-individual oxygen isotopic ranges are up to ca. 2‰ (n = 4), whereas in the Mesolithic, this increases to approximately 2–3‰ (n = 6)–a statistically significant shift (p = 0.005). In contrast, there is no statistically significant change for caprid intra-individual oxygen isotopic ranges through time: caprid intra-individual oxygen isotopic ranges are ~4–6‰ (n = 4) for the Late Upper Paleolithic, 2.5 to 5‰ (n = 3) in the Mesolithic, and 2.5 to 5‰ (n = 5).for the Neolithic. Based on both “population” scale (all observed δ^18^O values for all intra-tooth samples of each taxon within a specific time period) and ranges in δ^18^O values of individuals, the two species do not significantly overlap in degree of variability, ranges, or mean values of δ^18^O. There also does not appear to be a taxon-specific difference in variability, ranges, or mean values of δ^13^C.

### Variation in Teeth

**Fig 7:**
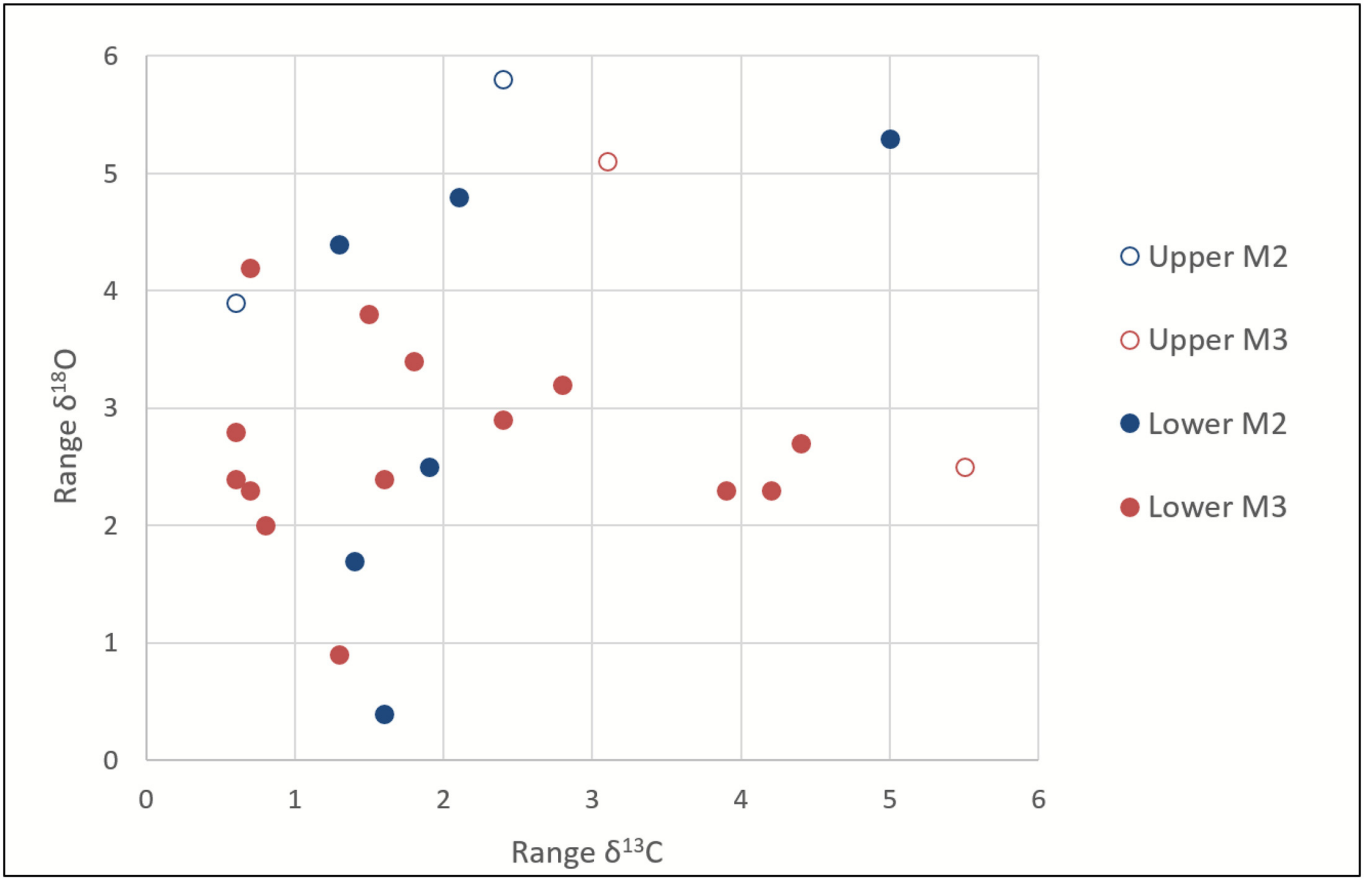
Scatter plot of ranges of δ^18^O and δ^13^C values in cervids and caprids by upper and lower molars showing no relationship between tooth sampled and δ^18^O and δ^13^C values.

Because different teeth (M2 vs. M3) form over different lengths of time (i.e. record different periods [36]) and have different crown heights, we evaluated whether δ^18^O and δ^13^C results in our sample might vary along these parameters and found no pattern that suggested intra-individual variation was strongly constrained by type of tooth (e.g. M2 vs M3) in this study (Fig 7). We found a weak yet statistically significant correlation between tooth crown height and range of δ^18^O values recorded within an individual (r^2^ = 0.22, p = 0.02). This relationship suggests that shorter crown heights record a smaller range of values, but explains only a small portion (22%) of the observed variation. A small degree of influence of crown height on the recorded range was expected; as noted above, a crown height of at least 18mm in caprid teeth was desirable in sample selection since this is likely to include the complete range of δ^18^O values recorded in the tooth (cf. [36]). Although it is necessary to sample across red deer M2-M3 pairs to detect the full to seasonal cycle of δ^18^O values [37], the M2 and M3 mineralize over a similar number of months [89]; thus, the amount of intra-tooth variation should not be influenced by type of tooth despite the signal being seasonally biased.

## Discussion and Conclusions

### Variation in δ^18^O and δ^13^C through time

That there are different isotopic patterns between the taxonomic categories, and within the taxa through time, suggests that there is not an overall change in the variability of environmental seasonality recorded in the teeth. Caprids act as a control group, preserving the same degree of intra-tooth variability throughout the Pleistocene-Holocene transition. Red deer intra-individual oxygen isotope ranges are significantly different between the Pleistocene/Late Upper Paleolithic and the Holocene/Mesolithic. The temporal difference in δ^18^O variability combined with the limited intra-tooth variability seen in red deer individuals suggests that variability is not wholly determined by factors such as tooth formation or species-specific physiology and instead may have to do with changes in migratory behavior and range size through time, as discussed above. There is no significant change in variability of δ^18^O ranges exhibited in caprids, suggesting seasonal mobility is likely restricted throughout time for these taxa. Furthermore, variability in carbon isotope ranges appears to have no relationship with taxa or time periods and is likely indicative of individual foraging preferences. Based on these results, we interpret that the data indicate red deer appear to have been migrating seasonally in the late Pleistocene/Late Upper Paleolithic, and potentially moving over smaller ranges in the early Holocene/Mesolithic. The combined caprid data suggest that these animals appear to not be migrating at any point in time and may have been a stable year round resource.

### Implications for archaeology

The nature of the archaeological record and the unknown size of herds and their ranges in the past make it especially challenging to interpret seasonal migratory behavior from archaeological stable isotope data. The data presented here suggest that these ungulate taxa, which were significant components of prehistoric diet and are found in varying relative abundances through time at the case study sites [11–17, 52, 53, 55], had distinct mobility patterns. These would likely have been known to human groups and would have influenced human decisions about seasonal settlement and food choice.

Caprids and red deer were not the only taxa influencing human mobility and resource use. Other terrestrial ungulates such as wild boar, roe deer, and aurochs were important economic species [11–17, 52, 53, 55], but these taxa do not generally migrate on a seasonal basis over long distances, although wild boar may have extremely large home ranges [100, 101] and information about aurochs mobility is largely based on anecdotal historical evidence when the range of this species was already significantly reduced [102]. Modern roe deer generally have very small migratory ranges [103–105]. These three taxa may have been resources that were accessible year round, and they are all found in the faunal assemblages at the study sites [15–17].

The seasonal availability of these terrestrial mammal resources would have played a role in determining human mobility patterns. Above all the movements of the red deer must have been the most important to people in the Late Upper Paleolithic, as they are the dominant species in the faunal assemblages at Nugljanska and Pupićina. There is some evidence for a change in mobility of red deer during the Mesolithic, though this could be further supported with a larger dataset. Nevertheless, this change coincides with more heterogenous zooarchaeological assemblages at these sites [17], and suggests that a dietary shift could have been influenced in part by unpredictability in red deer mobility. Based on these data, there does seem to be evidence for a focus on seasonally migrating red deer in the Late Upper Paleolithic, and a broadening diet in the Mesolithic focusing on non-migratory, more seasonally predictable foodstuffs.

We have shown through the incremental isotopic analysis of carbonate from caprid and red deer tooth enamel that there are marked differences in ranges of variation of oxygen isotope values. These data suggest that red deer were seasonally migrating, making them available near the cave sites at only certain parts of the year. It can be argued that caprids inhabited more restricted habitats and did not migrate long distances. The differing seasonal availability of these and other terrestrial mammals, supplemented by molluscs and plant foods, would have played a significant part in determining human mobility strategies during the Pleistocene-Holocene transition in the context of rapid environmental change and sea level rise in the northeastern Adriatic.
